# Three LysM effectors of *Zymoseptoria tritici* collectively disarm chitin‐triggered plant immunity

**DOI:** 10.1111/mpp.13055

**Published:** 2021-04-01

**Authors:** Hui Tian, Craig I. MacKenzie, Luis Rodriguez‐Moreno, Grardy C. M. van den Berg, Hongxin Chen, Jason J. Rudd, Jeroen R. Mesters, Bart P. H. J. Thomma

**Affiliations:** ^1^ Laboratory of Phytopathology Wageningen University and Research Wageningen Netherlands; ^2^ Institute for Plant Sciences Cluster of Excellence on Plant Sciences (CEPLAS) University of Cologne Cologne Germany; ^3^ Department of Bio‐Interactions and Crop Protection Rothamsted Research Harpenden UK; ^4^ Institute of Biochemistry University of Lübeck Lübeck Germany; ^5^ Departamento de Biología Celular, Genética y Fisiología Universidad de Málaga Málaga Spain

**Keywords:** chitin, effector, LysM, virulence, *Zymoseptoria*

## Abstract

Chitin is a major structural component of fungal cell walls and acts as a microbe‐associated molecular pattern (MAMP) that, on recognition by a plant host, triggers the activation of immune responses. To avoid the activation of these responses, the Septoria tritici blotch (STB) pathogen of wheat, *Zymoseptoria tritici*, secretes LysM effector proteins. Previously, the LysM effectors Mg1LysM and Mg3LysM were shown to protect fungal hyphae against host chitinases. Furthermore, Mg3LysM, but not Mg1LysM, was shown to suppress chitin‐induced reactive oxygen species (ROS) production. Whereas initially a third LysM effector gene was disregarded as a presumed pseudogene, we now provide functional data to show that this gene also encodes a LysM effector, named Mgx1LysM, that is functional during wheat colonization. While Mg3LysM confers a major contribution to *Z. tritici* virulence, Mgx1LysM and Mg1LysM contribute to *Z. tritici* virulence with smaller effects. All three LysM effectors display partial functional redundancy. We furthermore demonstrate that Mgx1LysM binds chitin, suppresses the chitin‐induced ROS burst, and is able to protect fungal hyphae against chitinase hydrolysis. Finally, we demonstrate that Mgx1LysM is able to undergo chitin‐induced polymerization. Collectively, our data show that *Z. tritici* utilizes three LysM effectors to disarm chitin‐triggered wheat immunity.

## Graphical abstract

Three *Zymoseptoria tritici* chitin‐binding LysM effectors differentially contribute to virulence by suppressing chitin‐induced host defences or by shielding hyphae against host chitinases through polymerization.

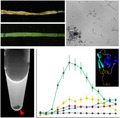

## 1 INTRODUCTION

Plants deploy an effective innate immune system to recognize and appropriately respond to microbial invaders. An important part of this immune system involves the recognition of conserved microbe‐associated molecular patterns (MAMPs) that are recognized by cell surface‐localized pattern recognition receptors (PRRs) to activate pattern‐triggered immunity (PTI) (Cook et al., 2015; Jones & Dangl, 2006; Thomma et al., 2001). PTI includes a broad range of immune responses, such as the production of reactive oxygen species (ROS), ion fluxes, callose deposition, and defence‐related gene expression (Altenbach & Robatzek, 2007; Boller & Felix, 2009; Jones & Dangl, 2006).

Chitin, a homopolymer of β‐(1,4)‐linked *N*‐acetylglucosamine (GlcNAc), is an abundant polysaccharide in nature and a major structural component of fungal cell walls (Free, 2013). Plants secrete hydrolytic enzymes, such as chitinases, as an immune response to target fungal cell wall chitin to disrupt cell wall integrity, and also to release chitin molecules that act as a MAMP that can be recognized by PRRs that carry extracellular lysin motifs (LysMs) to activate further immune responses against fungal invasion (Felix et al., 1993; Kombrink & Thomma, 2013; Sánchez‐Vallet et al., 2015). To date, chitin receptor complexes that comprise LysM‐containing receptors have been characterized in *Arabidopsis* and rice (Cao et al., 2014; Miya et al., 2007; Shimizu et al., 2010; Wan et al., 2012). Homologs of the crucial components of these complexes have also been identified in wheat (Lee et al., 2014).

To successfully establish an infection, fungal pathogens evolved various strategies to overcome chitin‐triggered plant immunity, such as alteration of cell wall chitin in such way that it is no longer recognized (Fujikawa et al., 2009, 2012), but also the secretion of effector proteins to either protect fungal cell walls against hydrolytic host enzymes or prevent the activation of chitin‐induced immunity (van den Burg et al., 2006; Kombrink et al., 2011; Marshall et al., 2011; Mentlak et al., 2012; Rovenich et al., 2014; Takahara et al., 2016). For example, some fungi can convert the surface‐exposed chitin in fungal cell walls to chitosan, which is a poor substrate for chitinases, thus avoiding the activation of chitin‐triggered immune responses during host invasion (El Gueddari et al., 2002; Ride & Barber, 1990). Furthermore, from the soilborne fungus *Verticillium dahliae* a secreted polysaccharide deacetylase was characterized to facilitate fungal virulence through direct deacetylation of chitin oligomers, converting them to chitosan (Gao et al., 2019). The use of effector molecules to successfully target chitin‐triggered plant immunity has been well‐studied for the tomato leaf mould fungus *Cladosporium fulvum*. This fungus secretes the invertebrate chitin‐binding domain (CBM14)‐containing effector protein Avr4 to bind fungal cell wall chitin, resulting in the protection of its hyphae against hydrolysis by tomato chitinases (van den Burg et al., 2006; van Esse et al., 2007). Additionally, *C. fulvum* secretes the effector protein Ecp6 (extracellular protein 6) that carries three LysMs, binds chitin, and suppresses chitin‐induced plant immunity. A crystal structure of Ecp6 revealed that two of its three LysM domains undergo ligand‐induced intramolecular dimerization, thus establishing a groove with ultrahigh (pM) chitin binding‐affinity that enables Ecp6 to outcompete plant receptors for chitin binding (Sánchez‐Vallet et al., 2013). Therefore, Avr4 and Ecp6 have complementary activities, where Avr4 protects hyphae but does not suppress chitin‐triggered immunity, and Ecp6 suppresses chitin‐triggered immunity but does not protect hyphae (Bolton et al., 2008; de Jonge et al., 2010). Homologs of Ecp6, coined LysM effectors, have been found in many fungi (de Jonge & Thomma, 2009) and include the functionally analysed *Magnaporthe oryzae* Slp1, *Colletotrichum higginsianum* Elp1 and Elp2, *V. dahliae* Vd2LysM, *Rhizoctonia solani* RsLysM and LysM1, and LysM2 from the mycoparasitic fungus *Clonostachys rosea* (Dolfors et al., 2019; Dubey et al., 2020; Kombrink et al., 2017; Mentlak et al., 2012; Takahara et al., 2016). In contrast, homologs of Avr4 are less widespread (Stergiopoulos et al., 2010).

*Zymoseptoria tritici* (formerly *Mycosphaerella graminicola*) is a host‐specific hemibiotrophic fungus and the causal agent of Septoria tritici blotch (STB) of wheat (*Triticum* spp.) (Eyal, 1999). On infection, wheat plants undergo an extended period of symptomless colonization of approximately 1 week, followed by the death of host tissues coinciding with rapid invasive growth and asexual reproduction of the fungus (Glazebrook, 2005; Kema et al., 1996; Pnini‐Cohen et al., 2000). This transition from biotrophic to necrotrophic growth of *Z. tritici* is associated with the induction of host immune processes such as a hypersensitive response (HR)‐like programmed cell death and differential expression of wheat mitogen‐activated protein kinase (MAPK) genes (Rudd et al., 2008). Three LysM effector genes were previously identified in the *Z. tritici* genome (Marshall et al., 2011). These comprise *Mg1LysM* and *MgxLysM*, which encode LysM effector proteins that carry a single LysM only, and *Mg3LysM* encoding an effector with three LysMs (Marshall et al., 2011). Whereas *Mg1LysM* and *Mg3LysM* were subjected to functional analysis, *MgxLysM* was disregarded because this gene lacked expressed sequence tag (EST) support and was believed to contain an intronic repeat insertion, rendering it a pseudogene. Both *Mg1LysM* and *Mg3LysM* were found to be induced during wheat infection, and both proteins were found to bind chitin. However, only Mg3LysM was found to suppress chitin‐induced plant immunity (Marshall et al., 2011). Surprisingly, and in contrast to Ecp6, both Mg1LysM and Mg3LysM were found to protect fungal hyphae against plant chitinase activity. Recently, a crystal structure was generated and revealed that Mg1LysM undergoes chitin‐dependent dimerization of ligand‐independent homodimers, and it was proposed that chitin‐induced polymerization of Mg1LysM in the fungal cell wall confers protection against chitinases (Sánchez‐Vallet et al., 2020). However, thus far the mechanism underlying the protection of cell walls by Mg3LysM remains unclear. In this study, we revisit the previously discarded *MgxLysM* gene and evaluate its contribution to *Z. tritici* virulence on wheat plants.

## 2 RESULTS

### 2.1 *Mgx1LysM* is expressed during wheat colonization

Although *MgxLysM* was previously reported to be a pseudogene and found not to be induced on wheat infection (Marshall et al., 2011), a more recent transcriptome profiling study on wheat demonstrated *MgxLysM* expression during host colonization, demonstrating that the initial assessment was incorrect (Rudd et al., 2015). Thus, we propose to rename MgxLysM as Mgx1LysM, according to the single LysM domain in the protein, similar to the previously described Mg1LysM effector (Marshall et al., 2011).

**FIGURE 1:**
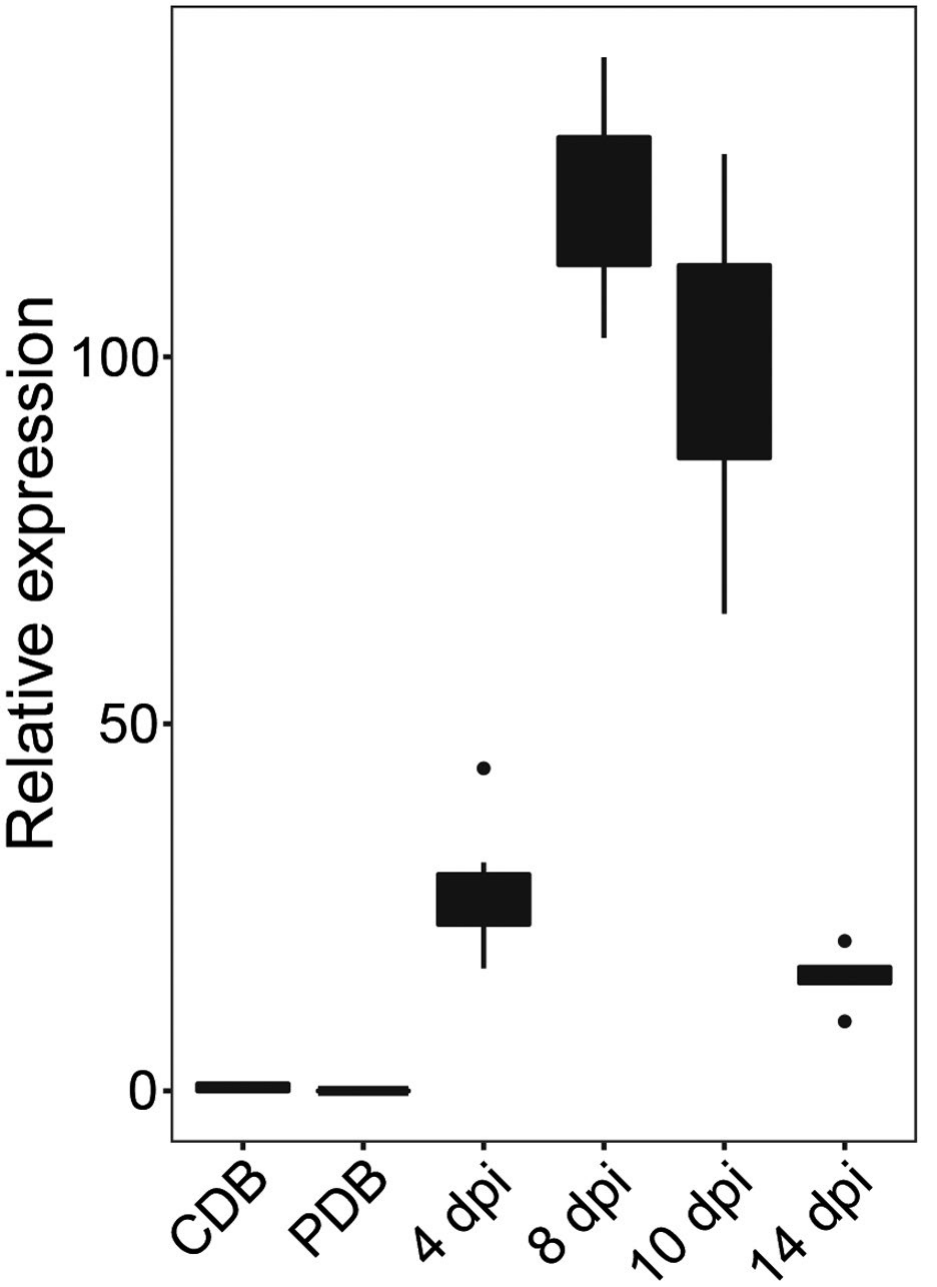
Expression of *Mgx1LysM* is induced in *Zymoseptoria tritici* on inoculation on wheat plants. Relative expression of *Mgx1LysM* at 4, 8, 10, and 14 days postinoculation (dpi) on wheat plants and on growth in vitro in Czapek‐Dox (CDB) or potato dextrose broth (PDB) when normalized to *Z. tritici β‐tubulin*. The boxplot was made with RStudio using the package ggplot2

To confirm the expression of *Mgx1LysM* in *Z. tritici* on host colonization, we inoculated the wild‐type strain IPO323 onto wheat leaves and sampled leaves at 0, 4, 8, 10, and 14 days postinoculation (dpi). In addition, we subjected IPO323 growing in vitro in Czapek‐Dox broth (CDB) and in potato dextrose broth (PDB) to expression analysis. We confirmed that *Mgx1LysM* is not expressed on growth in vitro, but only during host colonization at all tested time points (Figure 1). More specifically, *Mgx1LysM* expression was strongly induced at 4 dpi, peaked at 8 dpi, and dramatically decreased by 10 dpi. Interestingly, the peak of expression at 8 dpi is around the transition time when the infection switches from asymptomatic to symptomatic with the appearance of lesions on wheat leaves (Marshall et al., 2011).

### 2.2 Mgx1LysM contributes to *Z. tritici* virulence on wheat and displays functional redundancy with Mg1LysM and Mg3LysM

Because *Mgx1LysM* is expressed by *Z. tritici* during colonization of wheat plants, we further assessed whether Mgx1LysM contributes to *Z. tritici* virulence and whether it shares functional redundancy with Mg1LysM and Mg3LysM. To this end, we generated the single‐gene deletion mutant ∆*Mgx1*, the double‐gene deletion mutants ∆*Mg1‐*∆*Mgx1* and ∆*Mgx1*‐∆*Mg3*, and the triple‐gene deletion mutant ∆*Mg1‐*∆*Mgx1*‐∆*Mg3* in a ∆*ku70* mutant, which is unaltered in virulence but has improved homologous recombination frequencies (Bowler et al., 2010). The absence of *Mgx1LysM* combined with the presence of the resistance marker (*NAT*) was confirmed with PCR in two independent transformants for each of the combinations (Figure S1a). To assess the possibility of additional ectopic insertions, *NAT* copy numbers in the genomes of single‐, double‐, and triple‐gene deletion mutants were determined with quantitative real‐time PCR (qPCR), using *Z. tritici β‐tubulin* as a single gene‐copy control. As typically expected for *Agrobacterium tumefaciens*‐mediated transformation, all transformants carried a single copy of the transgene, suggesting a single T‐DNA insertion (Figure S1b).

**FIGURE 2:**
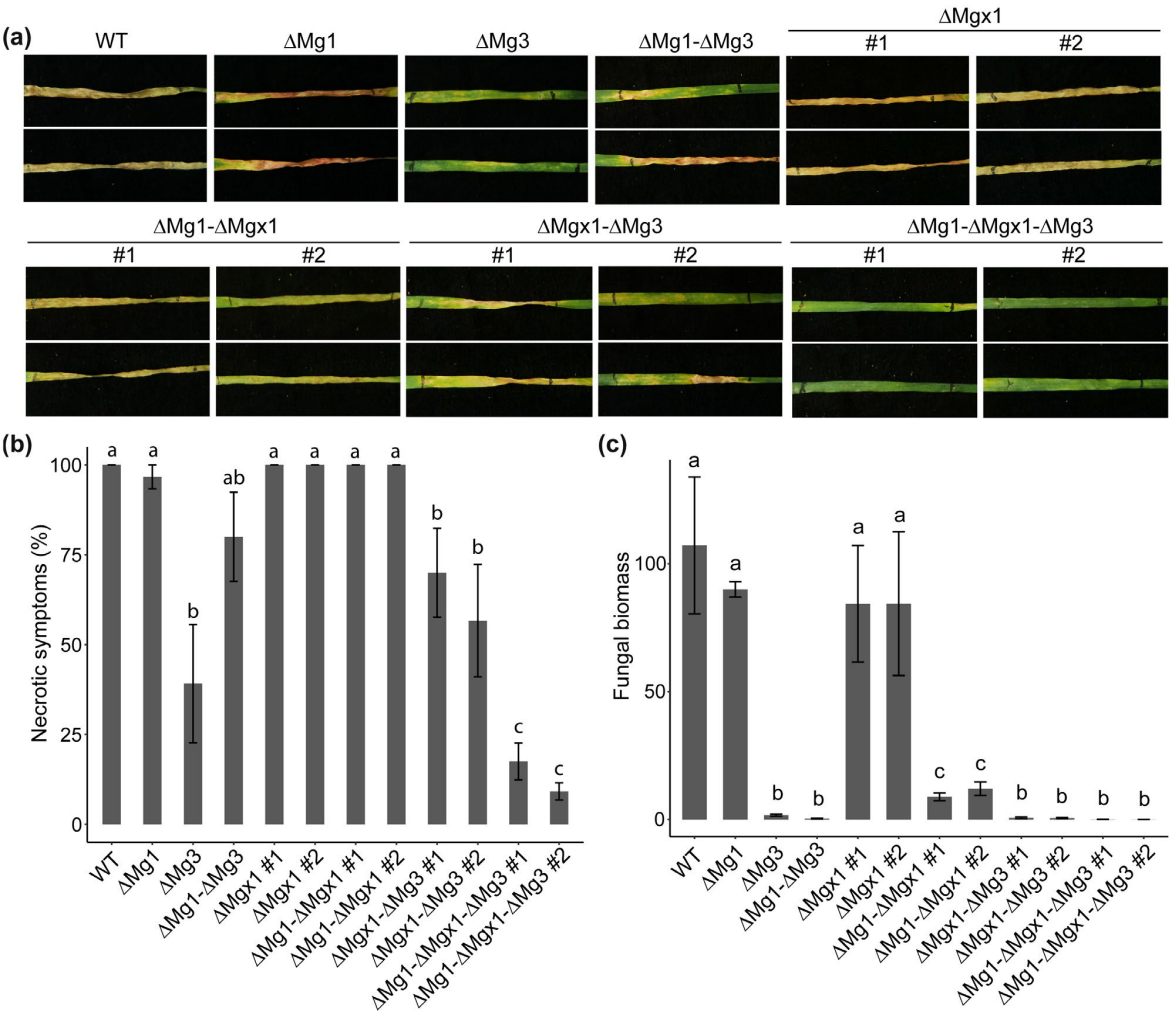
Mgx1LysM contributes to *Zymoseptoria tritici* virulence on wheat and displays functional redundancy with Mg1LysM and Mg3LysM. (a) Disease symptoms on wheat leaves at 21 days postinoculation (dpi) with the wild‐type strain (WT) and LysM effector gene deletion strains. (b) Quantification of the necrotic area on wheat leaves inoculated with WT and LysM effector gene deletion strains at 21 dpi. (c) Fungal biomass determined with quantitative PCR on *Z. tritici β‐tubulin* relative to the wheat cell division control gene, on wheat leaf samples harvested at 21 dpi. Graphs were made with RStudio using the package ggplot2 and different letters indicate significant differences between each inoculation, which were calculated with IBM Statistics 26 with one‐way analysis of variance (Duncan, *p* < .05). Fungal inoculation experiments were conducted on six plants with six first‐primary leaves per inoculation and repeated twice with similar results

To evaluate the contribution to virulence of each of the LysM effectors, two independent ∆*Mgx1*, ∆*Mg1*‐∆*Mgx1*, ∆*Mgx1‐*∆*Mg3*, and ∆*Mg1‐*∆*Mgx1‐*∆*Mg3* transformants were inoculated onto wheat plants, together with the previously tested gene deletion mutants ∆*Mg1,* ∆*Mg3*, and ∆*Mg1‐*∆*Mg3* (Marshall et al., 2011). All mutants were generated in the ∆*ku70* mutant that was used as wild‐type (WT) in this study. By 21 dpi, the WT strain caused typical necrosis symptoms on the wheat leaves, while ∆*Mg3* strains caused fewer necrotic symptoms (Figure 2a,b) as previously reported (Marshall et al., 2011). Furthermore, as previously reported, plants inoculated with the ∆*Mg1* strains developed similar necrosis as with the WT strain. We now show not only that the ∆*Mgx1* strains caused similar levels of necrosis as the WT and ∆*Mg1* strains, but also that the ∆*Mg1*‐∆*Mgx1* strains showed no apparent decrease in disease development, suggesting that these two LysM effectors are dispensable for virulence of *Z. tritici*. In line with these observations, both the ∆*Mgx1*‐∆*Mg3* strains and the ∆*Mg1*‐∆*Mg3* strains induced similar symptoms to the ∆*Mg3* strains (Figure 2a,b). Nevertheless, the necrotic symptoms caused by inoculation with the ∆*Mg1*‐∆*Mgx1*‐∆*Mg3* strains were drastically reduced when compared with those caused by the ∆*Mg3* strains. Collectively, these findings suggest that Mg3LysM is the most important LysM effector for *Z. tritici* disease development, and that Mgx1LysM and Mg1LysM contribute to disease development through redundant functionality.

To further substantiate the fungal colonization assessments, we measured fungal biomass with qPCR. While the ∆*Mg1* and ∆*Mgx1* strains developed similar amounts of fungal biomass as the WT strain, ∆*Mg1*‐∆*Mgx1* strains displayed significantly compromised colonization, but not as compromised as the ∆*Mg3* strains or the double and triple mutants that lack *Mg3LysM* (Figure 2c). These observations confirm that the three LysM effectors make differential contributions to symptom display, which is accompanied by distinct differential contributions to fungal colonization. Thus, our findings present evidence for partially redundant, but also partially divergent, contributions of the three LysM effectors to *Z. tritici* virulence.

### 2.3 LysM effectors differentially contribute to *Z. tritici* pycnidia formation on wheat

Soon after the onset of necrotic symptoms, asexual fruiting bodies (pycnidia) can be formed (Karisto et al., 2018; Kema et al., 1996). Thus, we determined the percentage of leaf surface displaying pycnidia coverage at 17 dpi. Surprisingly, repeated assays revealed that the ∆*Mg1* strains developed significantly more pycnidia than the WT strain, whereas the ∆*Mgx1* strains, like the ∆*Mg3* strains, produced no to only a few pycnidia (Figure S2). Accordingly, whereas the ∆*Mg1*‐∆*Mgx1* strains developed an intermediate number of pycnidia, all mutants that involved ∆*Mg3* were devoid of pycnidia (Figure S2). These data first of all suggest that symptom development does not correlate with fungal colonization levels as measured by pycnidia formation and, furthermore, that the three LysM effectors display differential roles in fungal colonization.

### 2.4 Mgx1LysM binds chitin and suppresses the chitin‐induced ROS burst

**FIGURE 3:**
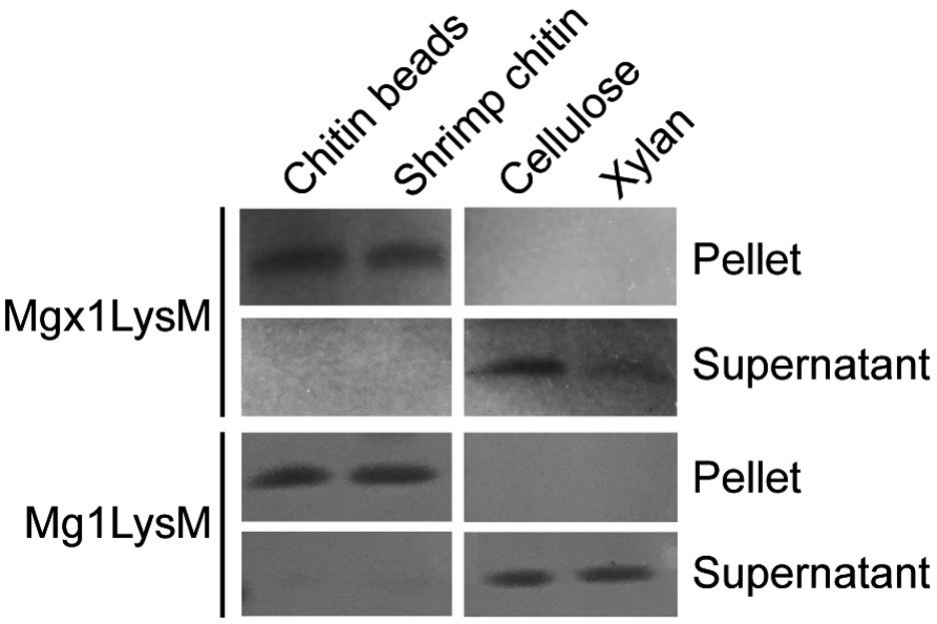
Mgx1LysM binds chitin. *Escherichia coli*‐produced Mgx1LysM and Mg1LysM were incubated with four chitin products for 6 hr and, after centrifugation, pellets and supernatants were analysed using polyacrylamide gel electrophoresis followed by Coomassie brilliant blue staining

To investigate how Mgx1LysM contributes to *Z. tritici* virulence during wheat colonization, we first assessed its substrate‐binding characteristics. Mgx1LysM was heterologously expressed in *Escherichia coli* and subjected to a polysaccharide precipitation assay. Mgx1LysM was incubated with chitin beads and shrimp shell chitin, but also with plant‐derived cellulose and xylan, revealing that Mgx1LysM binds chitin beads and shrimp shell chitin but not cellulose or xylan (Figure 3). Thus, Mgx1LysM resembles Mg1LysM that similarly binds chitin but not cellulose or xylan (Figure 3).

**FIGURE 4:**
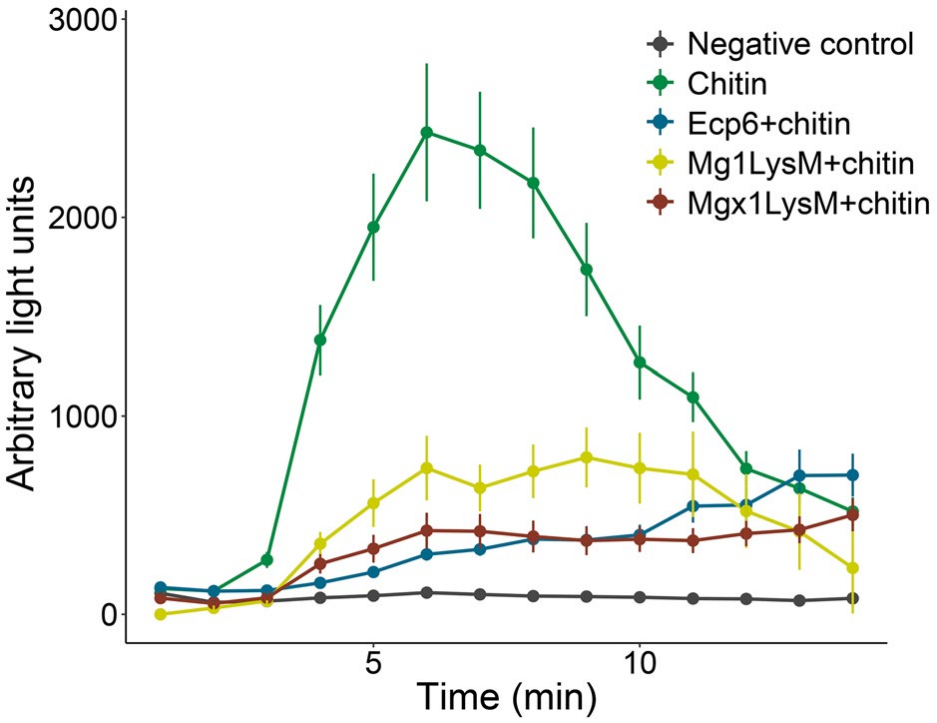
Mgx1LysM suppresses the chitin‐induced reactive oxygen species (ROS) burst. Leaf discs of *Nicotiana benthamiana* were treated with chitohexaose (chitin) to induce ROS production. Chitin was preincubated with Ecp6, Mg1LysM, or Mgx1LysM for 2 hr and subsequently added to the leaf discs. Error bars represent standard errors from five biological replicates

To test whether Mgx1LysM can prevent chitin‐triggered immunity in plants, the occurrence of a chitin‐induced ROS burst was assessed in *Nicotiana benthamiana* leaf discs on treatment with 10 μM chitohexaose (chitin) in the presence or absence of effector protein. As previously demonstrated (de Jonge et al., 2010), *C. fulvum* Ecp6 suppresses ROS production in this assay (Figure 4). Remarkably, preincubation of 10 μM chitin with 50 μM Mgx1LysM prior to the addition to leaf discs led to a significant reduction of the ROS burst (Figure 4), demonstrating its ability to suppress chitin‐induced plant immune responses. This finding was unexpected because we previously found that its close homolog Mg1LysM cannot suppress a chitin‐induced defence response in a tomato cell culture (Marshall et al., 2011), albeit that in that study Mg1LysM was heterologously produced in the yeast *Pichia pastoris* rather than in *E. coli*. To revisit this initial observation, we now test whether *E. coli*‐produced Mg1LysM is able to suppress the chitin‐induced ROS burst. Indeed, similar to the results obtained for Mgx1LysM, we observed that preincubation of 10 μM chitin with 50 μM Mg1LysM prior to the addition to leaf discs led to a significantly compromised ROS burst. Thus, both LysM effectors can suppress chitin‐triggered host immunity.

### 2.5 Mgx1LysM protects hyphae against chitinases

**FIGURE 5:**
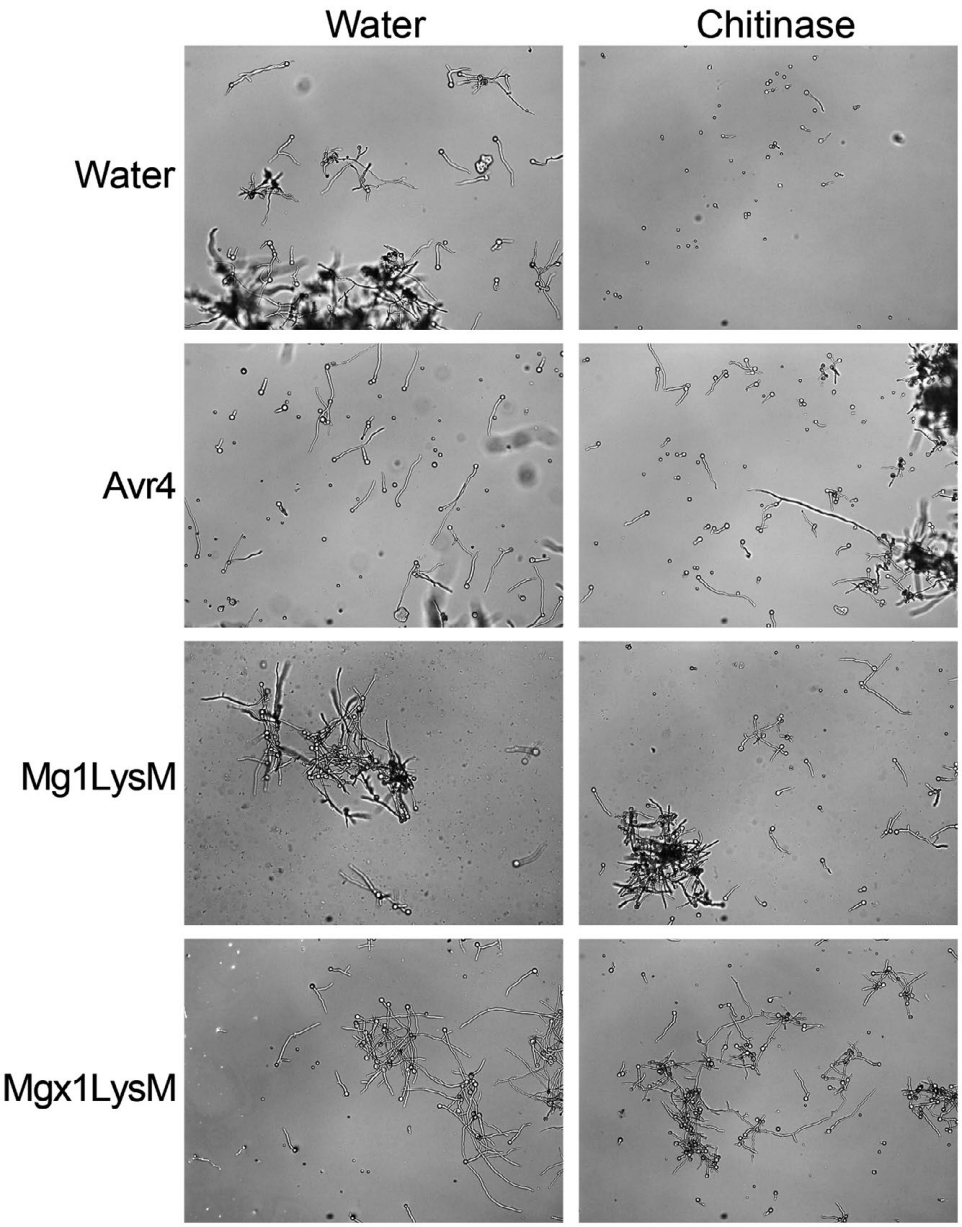
Mgx1LysM protects hyphal growth of *Trichoderma viride* against chitinase hydrolysis. Microscopic pictures of *T. viride* grown in vitro with or without 2 hr of preincubation with *Cladosporium fulvum* Avr4, or *Zymoseptoria tritici* Mg1LysM or Mgx1LysM, followed by the addition of chitinase or water. Pictures were taken c.4 hr after the addition of chitinase

We previously demonstrated that Mg1LysM can protect fungal hyphae against chitinase hydrolysis (Marshall et al., 2011). To evaluate a possible role in hyphal protection, Mgx1LysM was tested for its ability to protect hyphae of *Trichoderma viride*, a fungus that exposes its cell wall chitin in vitro, against chitinases (Mauch et al., 1988). The *C. fulvum* effector proteins Avr4 and Mg1LysM were used as positive controls based on their previously demonstrated ability to protect fungal hyphae (van den Burg et al., 2006; Marshall et al., 2011). As expected, while the addition of chitinase drastically inhibited *T*. *viride* hyphal growth, Avr4 as well as Mg1LysM protected the hyphae against hydrolysis by chitinases from *Clostridium thermocellum* (Figure 5) as well as from tomato (Figure S3). Furthermore, Mgx1LysM similarly protected the hyphae against chitinase hydrolysis (Figure 5).

### 2.6 Mgx1LysM undergoes chitin‐dependent polymerization

**FIGURE 6:**
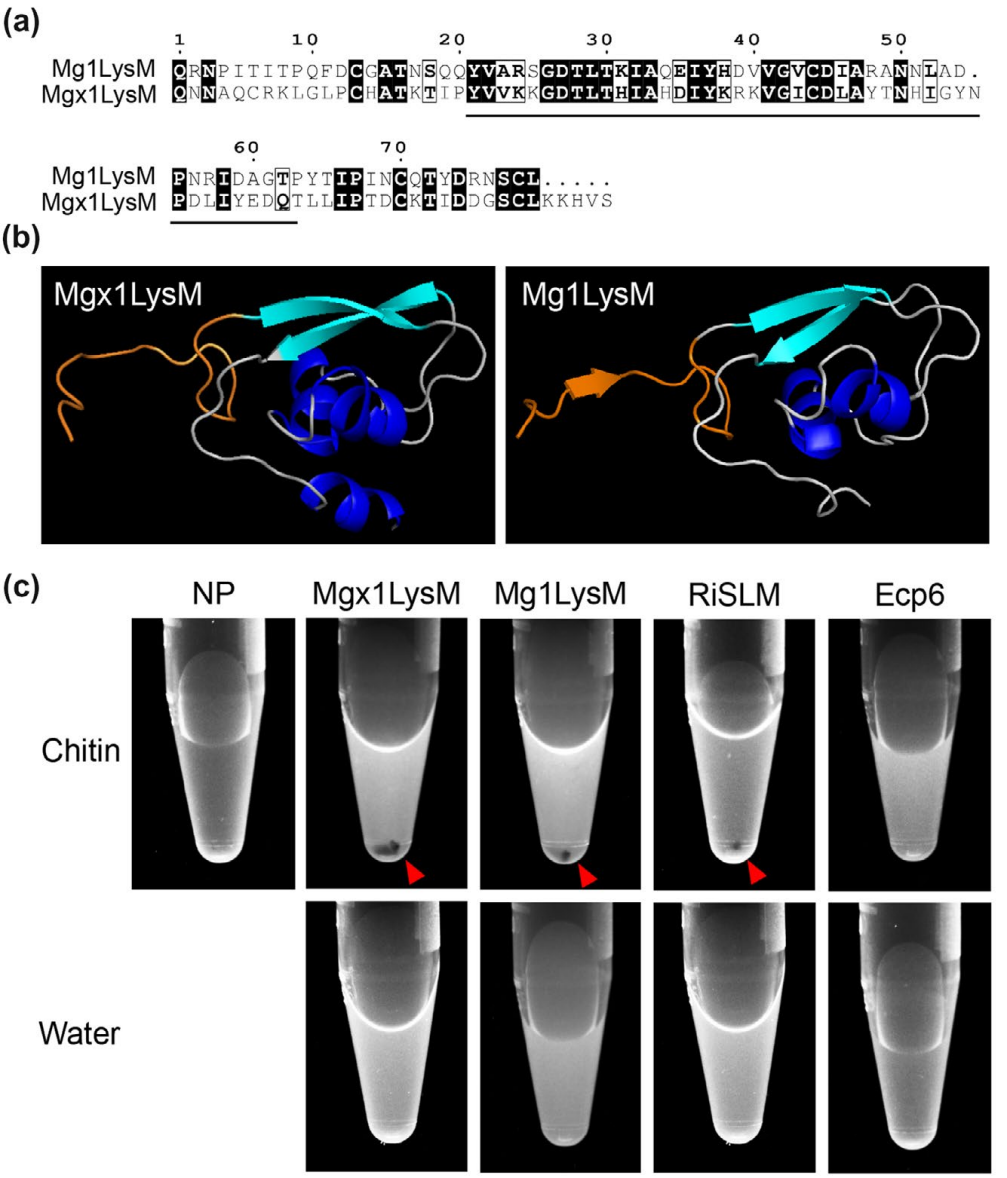
Mgx1LysM undergoes chitin‐induced polymerization. (a) Amino acid sequence alignment of Mgx1LysM and Mg1LysM. The LysM is indicated with black underlining. (b) I‐TASSER software‐based in silico prediction of the three‐dimensional structure of Mgx1LysM (left) based on the recently generated crystal structure of Mg1LysM (right) (Sánchez‐Vallet et al., 2020). The *N*‐terminal 15 amino acids of both proteins are depicted in orange. Structures are visualized using the PyMOL molecular graphics system (Schrodinger LLC, 2015). (c) The LysM effector Mgx1LysM, together with RiSLM and Mg1LysM as positive controls, and Ecp6 as negative control, were incubated with chitohexaose (chitin) or water. After overnight incubation, methylene blue was added and protein solutions were centrifuged, resulting in protein pellets (red arrowheads) as a consequence of polymerization for Mgx1LysM, Mg1LysM, and RiSLM, but not for Ecp6

Recently, Mg1LysM was demonstrated to protect fungal hyphae through chitin‐dependent polymerization of chitin‐independent Mg1LysM homodimers (Sánchez‐Vallet et al., 2020). To assess whether this trait is shared by Mgx1LysM, the amino acid sequence of Mgx1LysM was aligned with Mg1LysM, displaying an overall sequence identity of 44% (Figure 6a). As expected, the predicted three‐dimensional structure of Mgx1LysM shows a typical LysM fold with two antiparallel β‐sheets adjacent to two α‐helices (Figure 6b) (Bateman & Bycroft, 2000; Bielnicki et al., 2006; Liu et al., 2012; Sánchez‐Vallet et al., 2013, 2020). More importantly, similar to Mg1LysM, Mgx1LysM carries a relatively long N‐terminal sequence (Figure 6b). For Mg1LysM it was recently shown that this N‐terminal tail of a single monomer runs antiparallel with the tail of another Mg1LysM monomer, leading to the formation of ligand‐independent homodimers (Sánchez‐Vallet et al., 2020). Structural modelling of Mgx1LysM suggests that this LysM effector is also able to dimerize via its N‐terminal tail (Figure 6b).

Besides ligand‐independent dimerization, the crystal structure of Mg1LysM furthermore revealed chitin‐dependent dimerization of the ligand‐independent homodimers (Sánchez‐Vallet et al., 2020). Based on further biochemical evidence the occurrence of chitin‐induced polymeric complexes was demonstrated (Sánchez‐Vallet et al., 2020). Thus, to assess whether Mgx1LysM similarly undergoes chitin‐induced polymerization, a previously developed centrifugation assay was performed using *E. coli*‐produced Mgx1LysM (Sánchez‐Vallet et al., 2020), with Ecp6 as negative control and Mg1LysM and RiSLM as positive controls. Like Mg1LysM, RiSLM, a LysM effector from the arbuscular mycorrhizal fungus *Rhizophagus irregularis*, has also been shown to undergo chitin‐induced polymerization (Sánchez‐Vallet et al., 2020). After incubation with chitin, protein samples were centrifuged at 20,000 × g in the presence of 0.002% methylene blue to visualize the protein. Similar to Mg1LysM and RiSLM, a clear Mgx1LysM pellet emerged upon incubation with chitin, and not in the absence of chitin, while no chitin‐induced polymerization was observed for Ecp6 (Figure 6c). Collectively, our data indicate that Mgx1LysM, like Mg1LysM, undergoes not only chitin‐dependent dimerization, but also ligand‐independent dimerization through interactions at the N‐termini of Mgx1LysM monomers, leading to polymerization of the LysM effector protein in the presence of chitin.

## 3 DISCUSSION

In this study, we demonstrate that the previously disregarded LysM effector gene as a presumed pseudogene of the fungal wheat pathogen *Z. tritici*, *Mgx1LysM*, is a functional LysM effector gene that plays a role in *Z. tritici* virulence during infection of wheat plants. Like the previously characterized *Z. tritici* LysM effectors Mg1LysM and Mg3LysM, Mgx1LysM binds chitin (Figure 3), suppresses chitin‐induced ROS production (Figure 4), and can protect fungal hyphae against chitinase hydrolysis (Figure 5). Moreover, like Mg1LysM, Mgx1LysM polymerizes in the presence of chitin (Figure 6). Through these activities, Mgx1LysM makes a noticeable contribution to *Z. tritici* virulence on wheat plants (Figure 2).

Merely based on expression profile as well as biological activities, the three genes seem to behave in a similar fashion and complete redundancy could be expected. However, this is not what we observed in the mutant analyses, as these revealed that Mg3LysM confers the largest contribution, as targeted deletion of *Mg3LysM*, but not of *Mg1LysM* or *Mgx1LysM*, results in a noticeable difference in symptomatology. Moreover, even the simultaneous deletion of *Mgx1LysM* and *Mg1LysM* did not lead to compromised necrosis development, although deletion of these two genes from the *Mg3LysM* deletion strain in the triple mutant resulted in a further decrease of virulence. Thus, although it can be concluded that all three LysM effectors contribute to fungal virulence, these findings are suggestive of partially redundant and partially additive activities. This suggestion is further reinforced when assessing pycnidia development and fungal colonization data that demonstrate that single LysM effector deletions have significant effects on these traits. However, it presently remains unknown through which functional divergence these differential phenotypes are established.

The ability to protect fungal hyphae against chitinase hydrolysis that is shared by the three *Z. tritici* LysM effectors (Figure 5) has previously been recorded for some, but not all, LysM effectors from other fungal species as well. For example, although *V. dahliae* Vd2LysM and *R. irregularis* RiSLM can protect hyphae as well (Kombrink et al., 2017; Zeng et al., 2020), *C. fulvum* Ecp6, *C. higginsianum* ChElp1 and ChElp2, and *M. oryzae* MoSlp1 do not possess such activity (de Jonge et al., 2010; Mentlak et al., 2012; Takahara et al., 2016). Intriguingly, all LysM effectors that contain a single LysM characterized to date (Mg1LysM, Mgx1LysM, RiSLM) were found to protect fungal hyphae. However, among the ones with two LysM domains members are found that do (Vd2LysM) and that do not (ChElp1, ChElp2, MoSlp1) protect, which is also true for members with three LysMs (Mg3LysM versus Ecp6, respectively), suggesting that the ability to protect hyphae is not determined by the number of LysMs in the effector protein. Previously, a mechanistic explanation for the ability to protect fungal cell wall chitin has been provided for the *C. fulvum* effector protein Avr4 that acts as a functional homolog of LysM effectors that protect fungal hyphae, but that binds chitin through an invertebrate chitin‐binding domain (CBM14) rather than through LysMs (van den Burg et al., 2006). Intriguingly, Avr4 strictly interacts with chitotriose, but binding of additional Avr4 molecules to chitin occurs through cooperative interactions between Avr4 monomers, which can explain the effective shielding of cell wall chitin (van den Burg et al., 2004). Despite being a close relative of *C. fulvum* in the Dothidiomycete class of ascomycete fungi, *Z. tritici* lacks an Avr4 homolog (Stergiopoulos et al., 2010). This may explain why the *Z. tritici* LysM effectors, in contrast to *C. fulvum* Ecp6, evolved the ability to protect fungal cell wall chitin. Recently, it has been proposed that the hyphal protection by LysM effectors that contain only a single LysM, including Mg1LysM and RiSLM, is due to chitin‐induced polymerization, leading to contiguous LysM effector filaments that are anchored to chitin in the fungal cell wall to protect these cell walls (Sánchez‐Vallet et al., 2020). Here, we show that Mgx1LysM similarly undergoes chitin‐induced polymer formation (Figure 6).

It was previously reported that Mg1LysM was incapable of suppressing chitin‐induced immune responses (Marshall et al., 2011), in contrast to the immune‐suppressive activity of Mg3LysM. A mechanistic explanation for this observation was found in the observation that Ecp6, being a close homolog of Mg3LysM, was able to efficiently sequester chitin oligomers from host receptors through intramolecular LysM dimerization, leading to a binding groove with ultrahigh chitin‐binding affinity. As a single LysM‐containing effector protein, Mg1LysM lacks the ability to undergo intramolecular LysM dimerization, and thus to form an ultrahigh affinity groove for chitin binding, which could explain the inability to suppress immune responses by out‐competition of host receptor molecules for chitin binding. However, this mechanistic explanation was recently challenged by data showing that the *R. irregularis* RiSLM is able to suppress chitin‐triggered immunity as well (Zeng et al., 2020). In the present study we show not only that Mgx1LysM can suppress chitin‐triggered immunity, but also that Mg1LysM possesses this activity (Figure 4). However, it needs to be acknowledged that, whereas we used *P*. *pastoris*‐produced protein in our initial analyses (Marshall et al., 2011), we used *E. coli*‐produced protein in the current study. More recent insights after the publication of our initial study have revealed that LysM effector proteins may bind chitin fragments that are released from the *P. pastoris* cell walls during protein production, which may compromise the activity of the protein preparation in subsequent assays (Kombrink et al., 2017; Sánchez‐Vallet et al., 2013, 2020). As the *E. coli* cell wall is devoid of chitin, partially or fully inactive protein preparations due to occupation of the substrate binding site are unlikely to occur. However, because Mg1LysM, Mgx1LysM, and RiSLM are able to suppress chitin‐triggered immunity, a mechanistic explanation needs to be provided for the suppressive activity that does not involve substrate sequestration purely based on chitin‐binding affinity. Possibly, these LysM effectors are able to perturb the formation of active chitin receptor complexes by binding to receptor monomers in a similar fashion as has been proposed for LysM2 of Ecp6 (Sánchez‐Vallet et al., 2013, 2015) to prevent the activation of chitin‐triggered immune responses. Alternatively, precipitation of polymeric complexes formed by LysM effectors and released chitin oligosaccharides may provide a mechanism to eliminate these oligosaccharides and prevent their interaction with host receptor molecules.

## 4 EXPERIMENTAL PROCEDURES

### 4.1 Gene expression analysis

Total RNA was isolated using the RNeasy Plant Mini Kit (Qiagen). For each sample, 2 µg of RNA was used for cDNA synthesis with M‐MLV reverse transcriptase (Promega) and 1 µl of the obtained cDNA was used for qPCR with SYBR Green master mix (Bioline) on a C1000 Touch thermal cycler (Bio‐Rad). Expression of *Mgx1LysM* was normalized to the *Z. tritici* housekeeping gene *β‐tubulin* using primer pairs *Mgx1LysM*‐F/*Mgx1LysM*‐R and *ZtβTUB*‐F1/R1, respectively (Table S1). Relative expression was calculated with the E^−∆^ *^C^* ^t^ method and the boxplot was made with RStudio using the package of ggplot2 (R Core Team, 2017; Wickham, 2016).

### 4.2 Heterologous protein production in *E. coli*

Signal peptide prediction was performed using SignalP v. 5.0 (http://www.cbs.dtu.dk/services/SignalP/). The coding region for the mature Mgx1LysM protein was amplified from *Z. tritici* IPO323 genomic cDNA using primers *Mgx1LysM*‐cDNA‐F/ R (Table S1) and cloned into the pETSUMO vector and transformed as pETSUMO‐Mgx1LysM into *E. coli* Origami for heterologous protein production as a fusion protein with a 6 × His‐SUMO affinity‐tag. *Mgx1LysM* expression was induced with 0.2 mM isopropyl β‐d‐1‐thiogalactopyranoside (IPTG) at 28 °C overnight. Next, *E. coli* cells were harvested by centrifugation at 3,800 × g for 1 hr and resuspended in 20 ml of cell lysis buffer (50 mM Tris‐HCl pH 8.5, 150 mM NaCl, 2 ml glycerol, 120 mg lysozyme, 40 mg deoxycholic acid, 1.25 mg DNase I and 1 protease inhibitor pill) and incubated at 4 °C for 2 hr with stirring, and centrifuged at 20,000 × g for 1 hr. The resulting cleared supernatant was immediately placed on ice and subjected to further purification.

The His60 Ni Superflow Resin (Clontech) was used for Mgx1LysM purification and first equilibrated with wash buffer (50 mM Na_2_HPO_4_, 150 mM NaCl, 10 mM imidazole, pH 8.0) after which the protein preparation was loaded on the column. The target protein was eluted with elution buffer (50 mM Na_2_HPO_4_, 150 mM NaCl, 300 mM imidazole, pH 8.0), and the purity of the elution was tested on a sodium dodecyl sulphate (SDS) polyacrylamide gel followed by Coomassie brilliant blue (CBB) staining. The 6 × His‐SUMO affinity‐tag was cleaved with the SUMO Protease ULP1 during overnight dialysis against 200 mM NaCl. Noncleaved Mgx1LysM fusion protein was removed using His60 Ni Superflow resin, and the flow‐through with cleaved Mgx1LysM was adjusted to the required concentration.

### 4.3 Chitin binding assay

*E. coli*‐produced proteins were adjusted to a concentration of 30 µg/ml in chitin binding buffer (50 mM Tris pH 8.0, 150 mM NaCl) and 800 µl of protein solution was incubated with 50 µl of magnetic chitin beads or 5 mg crab shell chitin, cellulose, or xylan in a rotary shaker at 4 °C for 6 hr. The insoluble fraction was pelleted by centrifuging at 13,500 × g for 5 min and resuspend in 100 µl of demineralized water. Supernatants were collected into Microcon Ultracel YM‐10 tubes (Merck) and concentrated to a volume of approximately 100 µl. For each of the insoluble carbohydrates, 30 µl of the pellet solution and the concentrated supernatant was incubated with 10 µl of SDS‐polyacrylamide gel electrophoresis (PAGE) protein loading buffer (4×; 200 mM Tris‐HCl, pH 6.5, 0.4 M dithiothreitol, 8% SDS, 6 mM bromophenol blue, 40% glycerol) and incubated at 95 °C for 10 min. Samples were loaded into an SDS‐polyacrylamide gel followed by CBB staining.

### 4.4 Hyphal protection against chitinase hydrolysis

*T. viride* conidiospores were harvested from 5‐day‐old potato dextrose agar (PDA; Oxoid), washed with sterile water, and adjusted to a concentration of 10^6^ spores/ml with potato dextrose broth (PDB; Becton Dickinson). Conidiospore suspensions were dispensed into a 96‐well microtitre plate in aliquots of 50 µl and incubated at room temperature overnight. Effector proteins were added to a final concentration of 10 µM, and after 2 hr of incubation 3 µl of chitinase from *Clostridium thermocellum* (Creative Enzymes) or 10 µl of crude extract of tomato chitinases was added into the appropriate wells. As control, sterile water was added. All treatments were further incubated for 4 hr and hyphal growth was inspected with a Nikon H600L microscope.

### 4.5 Reactive oxygen species measurement

Reactive oxygen species (ROS) production measurements were performed using three *N. benthamiana* leaf discs (Ø = 0.5 cm) per treatment, which were collected from 2‐week‐old *N. benthamiana* plants, placed into a 96‐well microtitre plate, and rinsed with 200 µl of demineralized water. After 24 hr the water was replaced by 50 µl of fresh demineralized water and the plate was incubated for another 1 hr at room temperature. Meanwhile, mixtures of (GlcNAc)_6_ (IsoSep AB) and effector proteins were incubated for 2 hr. In total, 20 µl of (GlcNAc)_6_ was added in a final concentration of 10 µM to trigger ROS production in the absence or presence of 100 µl of effector protein in a final concentration of 50 µM in a measuring solution containing 100 µM/L012 substrate (Fujifilm) and 40 µg/ml horseradish peroxidase (Sigma‐Aldrich). Chemiluminescence measurements were taken every minute over 30 min in a CLARIOstar microplate reader (BMG LABTECH).

### 4.6 *Agrobacterium tumefaciens*‐mediated *Z. tritici* transformation

To generate *Mgx1LysM* deletion mutants, approximately 1.0 kb upstream and 1.2 kb downstream fragments of *Mgx1LysM* were amplified from genomic DNA of *Z. tritici* IPO323 using primer pairs *Mgx1LysM*‐userL‐F/R and *Mgx1LysM*‐userR‐F/R (Table S1) and the amplicons were cloned into vector pRF‐NU2 as previously described (Frandsen et al., 2008). The resulting deletion construct was transformed into *Z. tritici* mutant ∆*ku70* and the previously generated ∆*Mg1LysM*, ∆*Mg3LysM*, and ∆*Mg1*‐∆*Mg3* to generate double‐ and triple‐gene deletion mutants. In short, minimal medium (MM) and induction medium (IM) were prepared at a pH of 7.0 and *Z. tritici* conidiospores were collected, washed, and adjusted to a final concentration of 10^7^ spores/ml. Transformation plates were incubated at 16 °C in the dark for 2–3 weeks. Putative transformants were transferred to PDA plates supplemented with 200 µg/ml cefotaxime and 25 µg/ml nourseothricin (Sigma‐Aldrich) and absence of *Mgx1LysM* was confirmed with PCR using the gene‐specific primers *Mgx1LysM*‐F/*Mgx1LysM*‐R and the primers *NAT*‐F/R (Table S1).

### 4.7 Gene copy number determination

The copy number of the *NAT* resistance marker gene was determined with qPCR using primers *NAT*‐F/R and *ZtβTUB*‐F2/R2 (Table S1). The primer efficiency was determined using a series of 10 × dilutions of the genomic DNAs extracted from the triple‐gene deletion strain ∆*Mg1*‐∆*Mgx1‐*∆*Mg3* (392 ng/µl) and the wild‐type strain IPO323 (600 ng/µl). qPCR assays were performed with an annealing temperature of 66 °C at which the calculated efficiencies of both primer pairs approached 100%. The *C*_t_ value of *NAT* was normalized to the single‐copy gene *β‐tubulin* and calculated with the E^−∆^ *^C^* ^t^ method.

### 4.8 *Z. tritici* inoculations on wheat

For all inoculation assays, the wheat cultivar Riband was used. *Z*. *tritici* wild‐type strain IPO323 and the mutants were grown either on yeast extract peptone dextrose (YPD; 10 g of yeast extract/L, 20 g of peptone/L, 20 g of dextrose, and 15 g of agar/L) or in yeast glucose medium (YGM; 10 g of yeast extract/L, 30 g of glucose/L) supplemented with appropriate antibiotics at 16 °C with orbital shaking (100 rpm) for at least 5 days to obtain yeast‐like conidiospores that were used for plant inoculation. To this end, conidiospores were collected by centrifuging the suspensions at 2,000 × g for 5 min and adjusting to a final concentration of 10^7^ spores/ml with 0.5% Tween 20 for inoculation by brushing on adaxial and abaxial sides of primary leaves of 11‐day‐old wheat plants. The inoculated plants were covered in a plastic tent for 2 days to secure high humidity, after which the tent was opened in one side.

Fungal biomass was measured with qPCR using a C1000 Touch thermal cycler (Bio‐Rad) with the *Z. tritici*‐specific *β*‐*tubulin* primers *ZtβTUB*‐F1/R1 in combination with primers *TaCDC*‐F/R that target the constitutively expressed cell division control gene of wheat (Table S1). Relative fungal biomass was calculated with the E^−∆^ *^C^* ^t^ method and boxplots were made with RStudio using the package of ggplot2 (R Core Team, 2017; Wickham, 2016).

### 4.9 Protein structure prediction and polymerization assay

The three‐dimensional structure of Mgx1LysM was predicted with I‐TASSER server (Roy et al., 2010; Yang & Zhang, 2015). For chitin‐induced polymerization assay of LysM effectors, concentrations of Mgx1LysM, Mg1LysM, RiSLM, and Ecp6 were adjusted to 200 µM, and 200 µl of each protein was incubated with 200 µl of 2 mM chitohexaose (Megazyme), or 200 µl of water as control, at room temperature overnight. The next day, 2 µl of 0.2% methylene blue (Sigma‐Aldrich) was added and incubated for 30 min after which protein solutions were centrifuged at 20,000 × g for 15 min. Photographs were taken with a ChemiDoc MP system (Bio‐Rad) with custom setting for RFP.

## CONFLICT OF INTEREST

The authors declare no conflict of interest exists.

## AUTHORS CONTRIBUTIONS

H.T., L.R.M., J.R.M., and B.P.H.J.T. conceived the study. H.T., C.I.M., and L.R.M. designed experiments. H.T., C.I.M., and G.C.M.B. performed experiments. H.T. analysed data and wrote the manuscript. J.J.R. and H.C. provided experimental materials. J.R.M. and B.P.H.J.T. supervised the project. All authors discussed the results and contributed to the final manuscript.

## Supporting information


**FIGURE S1** Genotypic confirmation of the *Mgx1LysM* deletion strains of *Zymoseptoria tritici*. (a) Agarose gel electrophoresis of the PCR products amplified from genomic DNA of the *Z. tritici* wild‐type (WT) and mutant strains. Primers Mgx‐F/R and NAT‐F/R were used to confirm the absence of *Mgx1LysM* and presence of the *NAT* resistance marker gene. (b) The *NAT* copy number in the genome of WT and mutant strains was determined by normalizing to the single‐copy *β‐tubulin* (*ZtTUB*) gene with quantitative PCR. The copy number was calculated with the E^−∆^ *^C^* ^t^ method. The bar graph is made with RStudio with the package ggplot2

**FIGURE S2** Pycnidia formation determined on wheat leaf samples inoculated with the wild‐type *Zymoseptoria tritici* strain (WT) and gene deletion mutants, harvested at 17 postinoculation (dpi). The boxplot graph was made with RStudio using the ggplot2 package and different letters indicate significant differences between each inoculation, which were calculated with IBM Statistics 26 with one‐way analysis of variance (Duncan, *p* < .05)

**FIGURE S3** Mgx1LysM protects hyphal growth of *Trichoderma viride* against the hydrolysis by a crude extract of tomato leaves containing chitinases. Microscopic pictures of *T. viride* grown with or without preincubation with Mgx1LysM, followed by the addition of a crude extract of tomato leaves containing chitinases. Pictures were taken 4 hr after chitinase addition

**TABLE S1** Primers used in this study

## Data Availability

Data available on request from the authors.
