# Corrigendum

**DOI:** 10.1111/mpp.13271

**Published:** 2022-11-09

In Tian et al. (2021), published in *Molecular Plant Pathology* 22, 683–693, Figure 6c (p. 689) contained an inadvertent image duplication in the water control panel. This did not affect the experimental data or conclusions.

The correct version is provided here:

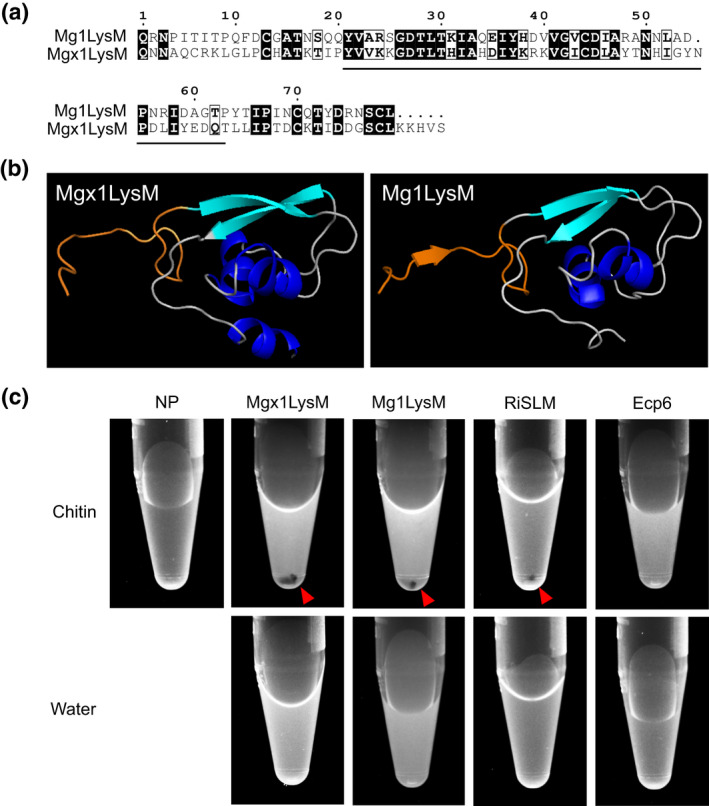

FIGURE 6 Mgx1LysM undergoes chitin‐induced polymerization. (a) Amino acid sequence alignment of Mgx1LysM and Mg1LysM. The LysM is indicated with black underlining. (b) I‐TASSER software‐based in silico prediction of the three‐dimensional structure of Mgx1LysM (left) based on the recently generated crystal structure of Mg1LysM (right) (Sánchez‐ Vallet et al., 2020). The N‐terminal 15 amino acids of both proteins are depicted in orange. Structures are visualized using the PyMOL molecular graphics system (Schrodinger LLC, 2015). (c) The LysM effector Mgx1LysM, together with RiSLM and Mg1LysM as positive controls, and Ecp6 as negative control, were incubated with chitohexaose (chitin) or water. After overnight incubation, methylene blue was added and protein solutions were centrifuged, resulting in protein pellets (red arrowheads) as a consequence of polymerization for Mgx1LysM, Mg1LysM, and RiSLM, but not for Ecp6
