# Supplementary material for: Three LysM effectors of Zymoseptoria tritici collectively disarm chitin‐triggered plant immunity
Source: Mol Plant Pathol. 2021 Apr 1;22(6):683–93. doi: 10.1111/mpp.13055 (PMC8126183; doi:10.1111/mpp.13055)
Supplement: Supplementary file 1 — FIGURE S1 Genotypic confirmation of the Mgx1LysM deletion strains of Zymoseptoria tritici. (a) Agarose gel electrophoresis of the PCR products amplified from genomic DNA of the Z. tritici wild‐type (WT) and mutant strains. Primers Mgx‐F/R and NAT‐F/R were used to confirm the absence of Mgx1LysM and presence of the NAT resistance marker gene. (b) The NAT copy number in the genome of WT and mutant strains was determined by normalizing to the single‐copy β‐tubulin (ZtTUB) gene with quantitative PCR. The copy number was calculated with the E−∆ C t method. The bar graph is made with RStudio with the package ggplot2 [file MPP-22-683-s003.docx]

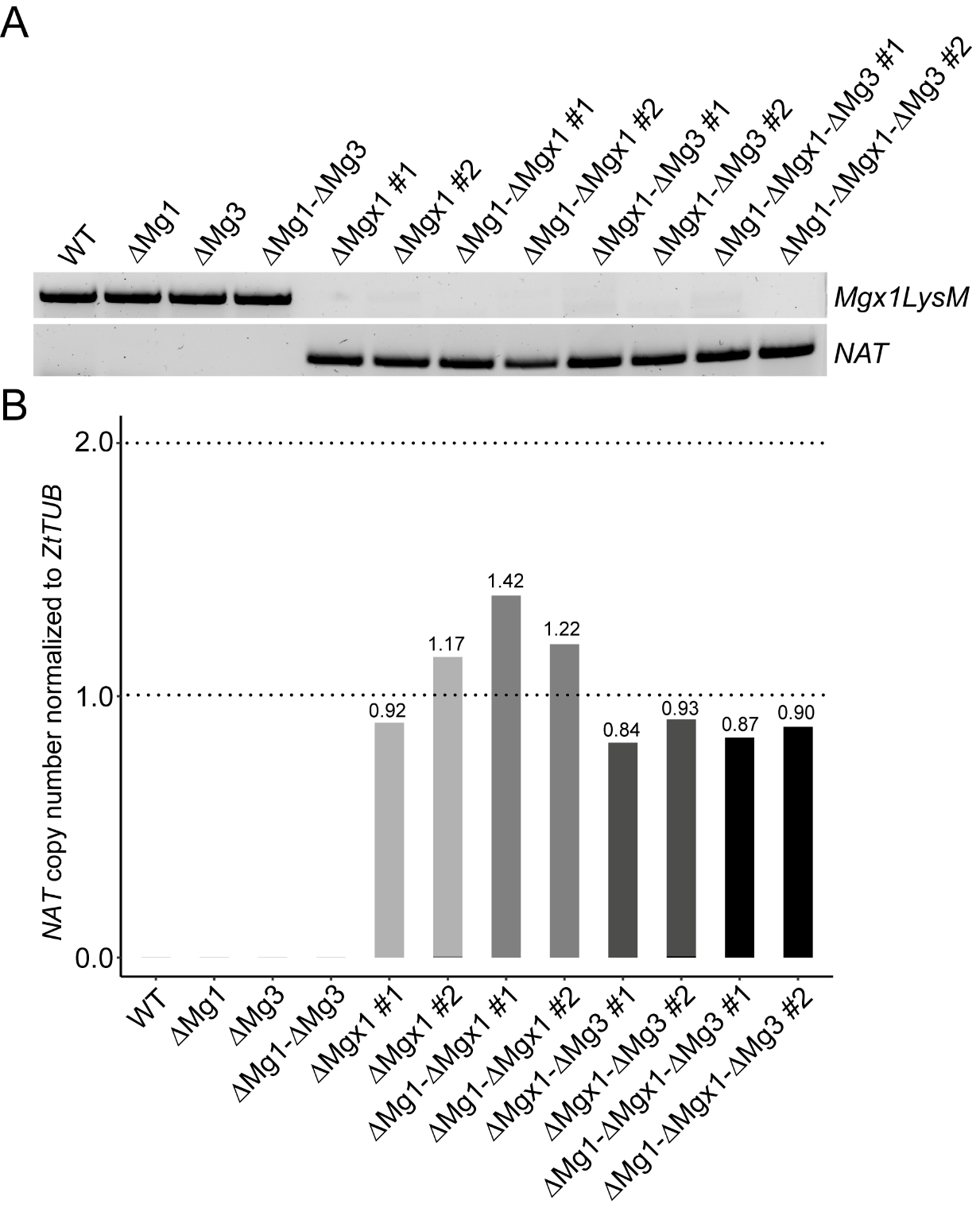


**Fig. S1** **Genotypic confirmation of the *Mgx1LysM* deletion strains of *Z. tritici*.** (A) Agarose gel electrophoresis of the PCR products amplified from genomic DNA of the *Z. tritici* wild-type (WT) and mutant strains. Primers Mgx-F/R and NAT-F/R were used to confirm the absence of *Mgx1LysM* and presence of the *NAT* resistance marker gene. (B) The *NAT* copy number in the genome of WT and mutant strains was determined by normalizing to the single-copy *β-tubulin* (*ZtTUB*) gene with real-time PCR. The copy number was calculated with the E^-∆Ct^ method. The bar graph is made with RStudio with the package ggplot2.
