# Supplementary material for: Three LysM effectors of Zymoseptoria tritici collectively disarm chitin‐triggered plant immunity
Source: Mol Plant Pathol. 2021 Apr 1;22(6):683–93. doi: 10.1111/mpp.13055 (PMC8126183; doi:10.1111/mpp.13055)
Supplement: Supplementary file 2 — FIGURE S2 Pycnidia formation determined on wheat leaf samples inoculated with the wild‐type Zymoseptoria tritici strain (WT) and gene deletion mutants, harvested at 17 postinoculation (dpi). The boxplot graph was made with RStudio using the ggplot2 package and different letters indicate significant differences between each inoculation, which were calculated with IBM Statistics 26 with one‐way analysis of variance (Duncan, p < .05) [file MPP-22-683-s001.docx]

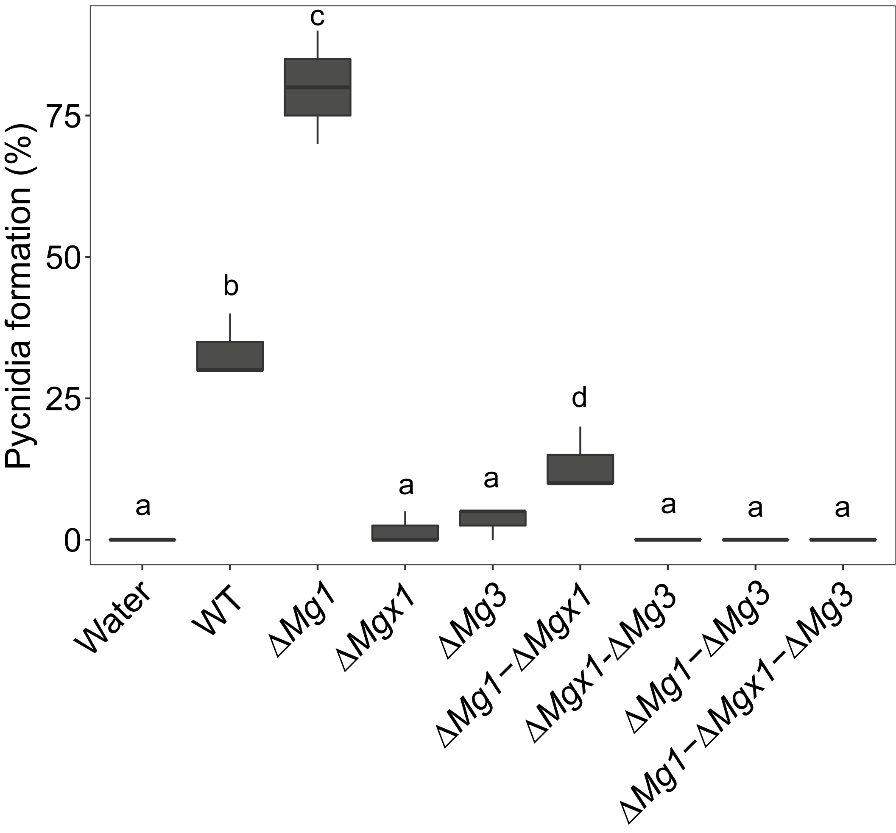


**Fig. S2** Pycnidia formation determined on wheat leaf samples inoculated with the wild-type *Z. tritici* strain (WT) and gene deletion mutants. harvested at 17 post inoculation (dpi). Boxplot graph was made with RStudio using the package of ggplot2 and different letters indicating significant differences between each inoculation were calculated with IBM Statistics 26 with One-way ANOVA (Duncan; P<0.05).
