# Supplementary material for: Three LysM effectors of Zymoseptoria tritici collectively disarm chitin‐triggered plant immunity
Source: Mol Plant Pathol. 2021 Apr 1;22(6):683–93. doi: 10.1111/mpp.13055 (PMC8126183; doi:10.1111/mpp.13055)
Supplement: Supplementary file 3 — FIGURE S3 Mgx1LysM protects hyphal growth of Trichoderma viride against the hydrolysis by a crude extract of tomato leaves containing chitinases. Microscopic pictures of T. viride grown with or without preincubation with Mgx1LysM, followed by the addition of a crude extract of tomato leaves containing chitinases. Pictures were taken 4 hr after chitinase addition [file MPP-22-683-s004.docx]

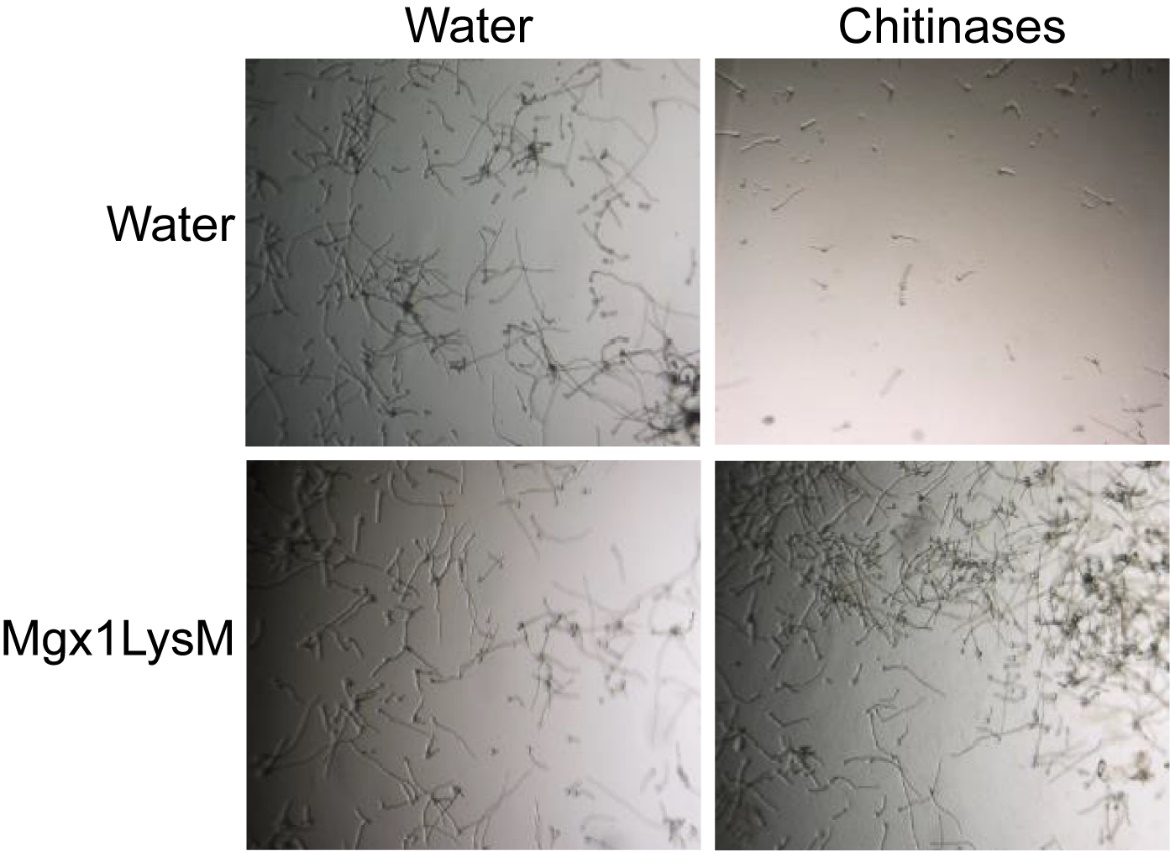


**Fig. S3 Mgx1LysM protects hyphal growth of *Trichoderma viride* against the hydrolysis by a crude extract of tomato leaves containing chitinases.** Microscopic pictures of *T. viride* grown with or without preincubation with Mgx1LysM, followed by the addition of a crude extract of tomato leaves containing chitinases. Pictures were taken 4 hours after chitinase addition.
