# Supplementary material for: Three LysM effectors of Zymoseptoria tritici collectively disarm chitin‐triggered plant immunity
Source: Mol Plant Pathol. 2021 Apr 1;22(6):683–93. doi: 10.1111/mpp.13055 (PMC8126183; doi:10.1111/mpp.13055)
Supplement: Supplementary file 4 — TABLE S1 Primers used in this study [file MPP-22-683-s002.docx]

**Table S1. Primers used in this study.**

| Primer name | Sequences |
| --- | --- |
| *Mgx1LysM*-userL-F | GGTCTTAAUAGAAAGGGCACTATTCAACAAGC |
| *Mgx1LysM*-userL-R | GGCATTAAUATACCATGCTGCCGAAGTTGAA |
| *Mgx1LysM*-userR-F | GGACTTAAUAAGGCGAAGCTCTTGAAAACTGG |
| *Mgx1LysM*-userR-R | GGGTTTAAUATCCAATCTTCACGTACCCGGTTTC |
| *Mgx1LysM*-F | CACGCCACGAAGACGATACCAT |
| *Mgx1LysM*-R | TTCAAGAGCTTCGCCTTTGGG |
| NAT-F | TTCCGCGTCACCGCCACCGGGGACGGCTTCACCCT |
| NAT-R | ACGCGCGCCCGACCCCG |
| *Mgx1LysM*-cDNA-F | GGTGGTGAATTCCAGAACAACGCACAGTGTCG |
| *Mgx1LysM*-cDNA-R | GGTGGTGCGGCCGCTTATTATCAGCTGACATGTTTCTTCAAG |
| *TaCDC*-F | CAAATACGCCATCAGGGAGAACATC |
| *TaCDC*-R | CGCTGCCGAAACCACGAGAC |
| *ZtβTUB*-F1 | AACGGTCGTTACCTCACCTG |
| *ZtβTUB*-R1 | ACGTTGTTCGGAATCCACTC |
| *ZtβTUB*-F2 | CTTCCGCAACGGTCGTTACCTCACCTGCTCC |
| *ZtβTUB*-R2 | CCCTCACCAGTGTACCAATGCAAGAAAGCC |
